# Feasibility and acceptability of home-based pulmonary rehabilitation for individuals with idiopathic pulmonary fibrosis in Delhi, India

**DOI:** 10.1177/14799731251375043

**Published:** 2025-09-03

**Authors:** Humaira Hanif, Obaidullah Ahmed, James R Manifield, Mohd Shibli, Amy Barradell, Zahira Ahmed, Dominic Malcolm, Andy Barton, Deepak Talwar, Mark W Orme, Sally J Singh

**Affiliations:** 1 Metro Centre for Respiratory Disease, Metro Hospital and Heart Institute, Noida, India; 2 School of Allied Health Sciences, Galgotias University, Greater Noida, India; 3 Department of Respiratory Sciences, 4488 University of Leicester, UK; 4 Centre for Exercise and Rehabilitation Science, NIHR Leicester Biomedical Research Centre-Respiratory, University Hospitals of Leicester, UK; 5 School of Sport, Exercise and Health Sciences, 5156 Loughborough University, Loughborough, UK

**Keywords:** feasibility, home-based pulmonary rehabilitation, idiopathic pulmonary fibrosis, interstitial lung disease, pulmonary rehabilitation, quality of life, qualitative research, self-management

## Abstract

**Objectives:**

To determine the feasibility and acceptability of home-based pulmonary rehabilitation (HBPR) for individuals with idiopathic pulmonary fibrosis (IPF).

**Methods:**

In this single-arm feasibility trial, individuals with IPF were recruited from Delhi, India, to a 6-weeks HBPR programme using a paper-based manual. Primary outcomes were feasibility (classified by ≥60% of eligible patients recruited and ≥70% of recruited patients completing the follow-up assessment) and intervention acceptability (semi-structured interviews).

**Results:**

Out of 42 screened, 36 individuals were eligible (86% of screened), and 30 were recruited (83% of eligible, 71% of screened; 60 ± 13 years, 53% female), with 25 completing their follow-up assessment (83% of recruited). HBPR was generally well-accepted, with qualitative themes including: ‘facilitators and barriers to HBPR’ (family support and flexibility of home environment were facilitators whereas lack of supervision and inability to follow a routine were barriers), ‘perceived changes from taking part in HBPR’ (improved exercise capacity, breathlessness, and independency), and ‘how to improve HBPR in the future’ (translating the manual into various languages, and incorporating into a more hybrid approach).

**Conclusion:**

HBPR using a paper-based manual was feasible and acceptable, potentially suitable for improving the uptake and completion of PR for individuals with IPF in Delhi, India.

## Introduction

The global adjusted prevalence of idiopathic pulmonary fibrosis (IPF) is estimated to be between 0.33 and 4.51 cases per 10,000 people.^1^ In India, IPF has an estimated national prevalence of 5.8–11.6 per 100,000.^2^ IPF is characterised by a progressive decline in lung function, associated worsening of dyspnoea, and has a poor prognosis.^3,4^ Furthermore, IPF is economically burdensome^5^ and is associated with reduced health-related quality of life (HRQoL), especially affecting domains of physical health.^6,7^

Evidence-based guidelines^8^ have provided strong recommendations from moderate quality evidence for the participation of adults with interstitial lung diseases (ILD) in pulmonary rehabilitation (PR) programmes to improve HRQoL and functional capacity, relying on evidence generated in high income countries. These guidelines have identified the need for further research to determine the optimal model of PR in this population (e.g., centre-based, home-based, telerehabilitation). The availability of PR in low-and middle-income countries (LMICs), including India, remains limited^9^ and alternative models may be attractive to providers and participants.^10,11^ Barriers to conventional, centre-based PR in LMICs, such as limited access, lack of infrastructure, family dependency, costs of travel, and household and/or employment responsibilities^10,12,13^ emphasize the need to develop a home-based PR (HBPR) model to address these barriers, and optimize uptake, compliance, and completion of PR in India.

A preference for the development of a paper-based manual, over an online or digital approach, to facilitate HBPR for individuals with IPF in Delhi, India has previously been identified as many reported not being digitally literate.^10^ Studies utilising this paper-based mode of PR for individuals with COPD in the UK (“Self-monitoring Programme of Activity Coping and Education for COPD: SPACEforCOPD©”) have found promising results relating to improved HRQoL, increased exercise capacity, and reduced dyspnoea.^14,15^

Due to both population and cultural differences between the UK and India, there is a need to develop and test a contextually-appropriate manual for individuals with IPF in Delhi which may improve the uptake to, and completion of, PR in this population. While initial findings in this area are promising,^16^ it is important to determine the feasibility and acceptability of PR, which conforms to guidelines on duration,^17^ before assessing clinical effectiveness.

This trial aimed to assess the feasibility and acceptability of a culturally-tailored, manual-based HBPR for individuals with IPF in Delhi, India.

## Methods

### Trial design

A single-arm mixed-methods feasibility trial was conducted. The trial was approved by the ethics review committees of Metro Hospitals & Heart Institute, Noida, India (ref: 62/MERB/2021; 10th February 2022) and University of Leicester, UK (ref: 31989; 13th March 2022), as part of the National Institute for Health and Care Research (NIHR) Global Health Research Group for Pulmonary Rehabilitation (Global RECHARGE) project.^18^

This trial was registered with the International Standard Randomized Controlled Trial Number (ref: ISRCTN14831771) and has been written following the Consolidated Standards of Reporting Trials (CONSORT) checklist for feasibility trials (Supplemental material A),^19^ the Template for Intervention Description and Replication (TIDieR) checklist (Supplemental material B),^20^ and the COnsolidated criteria for REporting Qualitative research (COREQ) checklist (Supplemental material C).^21^ All participants provided written informed consent.

### Setting

The trial was conducted in the Metro Centre of Respiratory Diseases (MCRD), Noida, Delhi, India. Recruitment occurred between 31st October 2022 to 5th January 2023.

### Participants

The inclusion criteria for this trial were adults aged ≥18 years, with a confirmed diagnosis of IPF (according to ATS/ERS guidelines),^22^ who were willing to provide informed consent. As the HBPR manual was written in English language, participants had to be able to understand basic English (defined as A1 or A2 level of the Common European Framework of Reference [CEFR] for Languages).^23^ Exclusion criteria were adults with comorbidities such as severe or unstable cardiovascular, other chronic conditions or locomotor difficulties that precluded exercise, malignant disease, or other serious illness which interfered with participation in the trial. Those ineligible for the trial were recorded on a trial screening log.

### Recruitment

Patients were referred by a pulmonologist at MCRD from outpatient clinics, or identified for the trial from medical records by a physiotherapist. The proposed sample size for the trial was 30. No formal sample size calculation was performed due to the feasibility nature of this trial; however, our proposed sample size was in line with previous feasibility trials.^24,25^

### Intervention

Our 6-weeks HBPR programme utilised a paper-based manual entitled “Self-monitoring Programme of Activity Coping and Education (SPACE) for ILD” (Figure S1) developed by researchers at the University of Leicester and MCRD, Delhi with expertise in pulmonary rehabilitation, and adapted from the ‘SPACEforCOPD©’ manual developed in the UK.^14,15^ Suggestions reported by individuals with IPF and caregivers regarding HBPR development in Delhi, India were incorporated during the development of this manual.^10^ This involved adding sections relating to goal setting, advice for carers, and contextually-appropriate case studies and dietary advice.

The manual contained the core elements of evidence-based PR (i.e., a programme of exercises and health education).^26,27^ The exercise programme consisted of individually prescribed walking, with diaries provided to monitor adherence and progression (Figure S2). Values achieved during walking tests at baseline along with walking goals (Figure S2) were added to the manual by the healthcare professional and participant at the end of the baseline assessment. Participants were instructed to walk at home around the same speed as during the baseline endurance shuttle walk test (ESWT). Participants were advised to perform resistance exercises of both upper and lower muscle groups (Figure S3) 3 times/week. Exercises were tailored for a low-resource environment by incorporating low-cost or self-made equipment (i.e., water bottles instead of dumbbells).

Self-management skills were promoted through goal-setting strategies, coping planning, and case studies. The manual included an action plan for exacerbation management. The researchers contacted the participants after 1 week of the intervention to determine their adherence and challenges related to HBPR. Following this period, participants were asked to contact healthcare workers (HCW; physiotherapists) if they experienced any further challenges or had any queries. Further details are provided in Supplemental material D.

### Data collection and management

Data were entered into the Research Electronic Data Capture (REDCap) web-based database.^28,29^ REDCap automated checks and 100% manual data checking were completed prior to locking the dataset for analysis. Interview data were collated in Microsoft Excel with quotes from transcripts, lists of codes, subthemes and themes.

### Primary outcome: Feasibility

Primary feasibility outcomes were recruitment and completion based on a predetermined traffic light system where green indicated the feasibility of the trial using the set methodology, amber indicated the need for modifications in the methodology, and red indicated the non-feasibility of the trial. The following thresholds were used: (i) recruitment (green ≥60%; amber 25%–59%; red <25% of eligible patients who were recruited) and (ii) completion (green ≥70%; amber 50%–69%; red <50% of those recruited who attended the discharge assessment).

### Acceptability

The acceptability of the intervention among participants was assessed by semi-structured interviews (interview schedule provided in Supplemental material E). Interviews were conducted by a physiotherapist (HH; female; MPT) with previous experience in qualitative data collection and analysis. Further support was provided by researchers at University of Leicester, UK. The purpose of the interview was explained to participants i.e., to understand their experiences of HBPR. Interviews were conducted face-to-face at MCRD or via telephone if in-person visits were not possible, and varied between English, Hindi, or combination of the two languages based on participant preference. No relationship between interviewer and participants were established prior to trial commencement. Field notes were taken during the interviews.

All interviews were audio recorded, transcribed verbatim and then translated into English to facilitate the analysis process. Translated quotes were checked against original transcripts by the interviewer (HH) to ensure meaning was maintained.

### Secondary outcomes

Data were collected in-person at MCRD and in accordance with the recommended minimum dataset for trials of PR in LMIC.^18^ Details of outcome measures and minimal clinically important differences (MCIDs) can be found in Supplemental material F. In brief, these included HRQoL (EuroQol Five Dimensions Five Levels [EQ-5D-5 L]^30^ and King’s Brief Interstitial Lung Disease [KBILD]),^31^ symptoms, (Medical Research Council [MRC],^32^ COPD Assessment Test [CAT],^33^ and Clinical COPD Questionnaire [CCQ]),^34^ psychological wellbeing (Hospital Anxiety and Depression scale [HADS],^35^ and physical capacity (Incremental and Endurance Shuttle Walk Tests [ISWT; ESWT],^36,37^ and 5x sit-to-stand).^38^

### Data analysis

#### Quantitative analysis

Data for baseline and follow-up time points were presented as descriptive statistics. Changes in secondary outcomes were benchmarked against previously published MCIDs (Supplemental material F).

#### Qualitative analysis

Template thematic analysis^39^ via the codebook approach was used to analyse qualitative data. Theories were generated via grounded theory and inductive reasoning. A six-phase process was conducted.^40^ (i) “Data familiarisation”: transcripts examined in detail by three researchers (HH, OA, JM); (ii) “Preliminary coding”: codes generated through an inductive approach and recorded in a codebook by one researcher (HH), (iii) “Generating initial themes”: patterns and relationship within and across the codes were discussed with three researchers (MO, JM, AB) and one researcher (HH) developed initial themes; (iv) “Developing and reviewing themes”: initial themes were discussed by four researchers (HH, AB, JM, MO) to ensure they told the story of the data; (v) “Refining, defining, and naming themes”: a detailed analysis of each theme was performed by four researchers (HH, AB, JM, MO) to decide on informative theme names and descriptions; (vi) “Writing up”: generated themes and illustrative quotes were contextualized, compared and contrasted to existing literature and the local context by one researcher (HH), with support from all authors.

## Results

### Feasibility: Participant screening and recruitment

**Figure 1.**
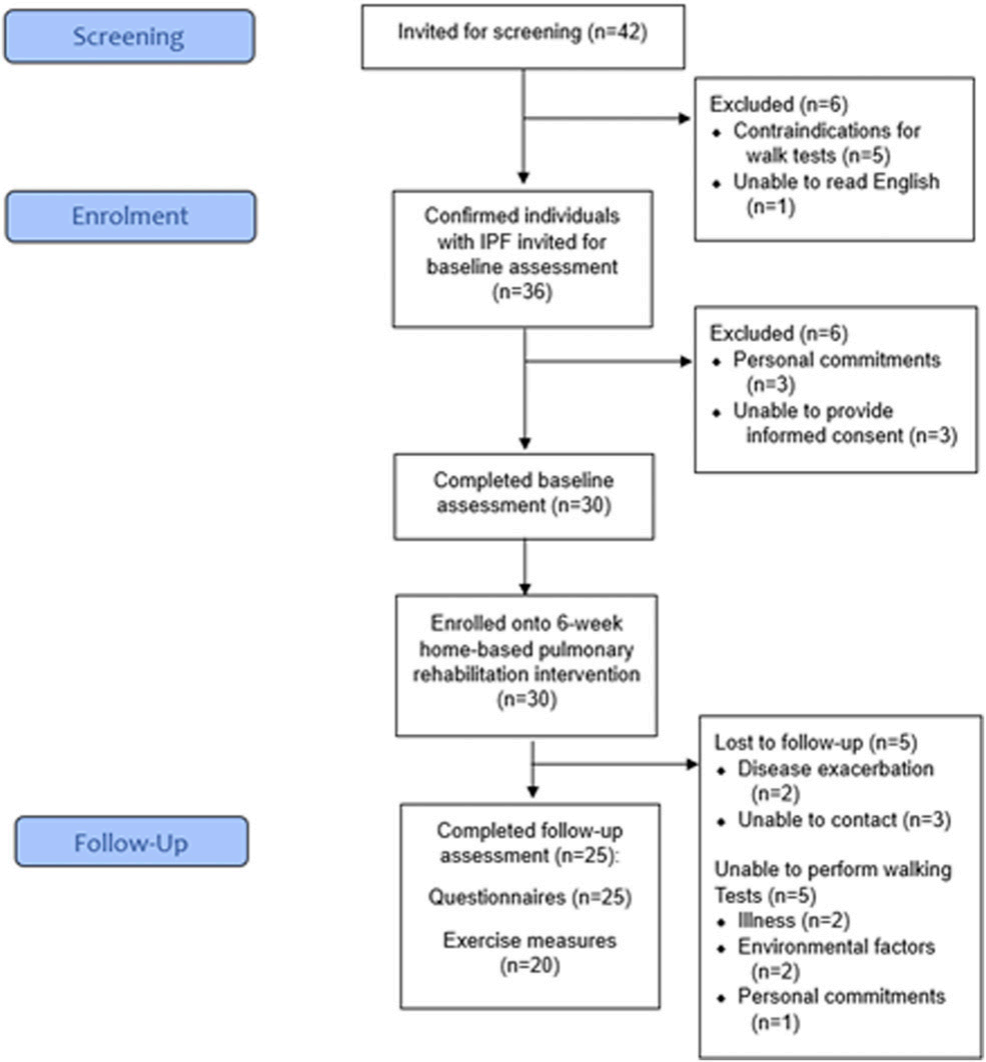
CONSORT diagram of the flow of participants through the trial.

Overall, 42 patients were screened of which 36 (86%) were eligible and invited to a baseline assessment (Figure 1). Reasons for ineligibility included contraindications for the walking tests (*n* = 5) and being unable to read English (*n* = 1). 30 participants (83% of those eligible) were recruited (Figure 1), with the main reason for declining participation being personal commitments (*n* = 3).

### Baseline characteristics

The mean (SD) age of participants was 59.9 (12.5) years and 53.3% were female (Table 1). Thirteen (43.3%) participants had a monthly household income greater than 67,500 rupees/month (equivalent to ∼808 USD). Most participants (70%) had no prior PR experience. Further details of baseline characteristics are presented in Supplemental material G.

**Table 1.** Baseline participant characteristics.

Characteristics	All participants (*n* = 30)
Demographics	
Age, years	59.9 (12.5)
Sex, *n*(%) female	16 (53.3)
Weight, kg	66.8 (9.5)
BMI	26.3 (3.8)
Age at leaving full-time education in years	20.5 (6.5)
Highest level of education, *n*(%)	
Incomplete primary education	2 (6.7)
Primary education	0 (0.0)
Secondary education	3 (10.0)
Higher secondary education	4 (13.3)
Graduation	12 (40.0)
Post-graduation	9 (30.0)
Employment status, *n*(%)	
In paid work (employed)	5 (16.7)
In paid work (self-employed)	3 (10.0)
In unpaid work	1 (3.3)
Not in work	21 (70.0)
Full-time homemaker or caregiver	13 (43.3)
Unemployed–able to work and seeking occupation	0 (0.0)
Unemployed–able to work and not seeking occupation	0 (0.0)
Unemployed–unable to work	0 (0.0)
Retired	8 (26.7)
Household income, *n*(%)	
Less than 14,000 (Rupees/month)	0 (0.0)
14001–21,500 (Rupees/month)	2 (6.7)
21,501–30,000 (Rupees/month)	1 (3.3)
30,001–37,000 (Rupees/month)	1 (3.3)
37,001–44,500 (Rupees/month)	3 (10.0)
44,501–52000 (Rupees/month)	5 (16.7)
52,001–60,000 (Rupees/month)	2 (6.7)
60,001–67,500 (Rupees/month)	3 (10.0)
Greater than 67,500 (Rupees/month)	13 (43.3)
Previous PR experience, *n* (%)	
Yes	9 (30.0)
No	21 (70.0)

*Note*: Data presented as mean (SD) unless otherwise stated.

### Feasibility: Participant completion

Out of the 30 recruited participants, 25 participants (83%) completed their follow-up assessment (Figure 1). Five participants (17%) withdrew due to disease exacerbation (*n* = 2, 7%) or being uncontactable (*n* = 3, 10%). Of the 25 completers, five participants (20%) completed their follow-up visit by telephone (rather than face-to-face) due to illness (*n* = 2, 8%), environmental factors (*n* = 2, 8%) or personal commitments (*n* = 1, 4%). The physical measures (i.e., ISWT, ESWT, and 5x sit-to-stand) at follow-up were therefore missing for these participants.

### Acceptability

Twenty-five interviews were conducted with recruited participants, either in person (*n* = 13 [52%]) or via telephone (*n* = 12 [48%]). The mean age of participants was 59.9 ± 12.5 years, 15 (60%) were female, and 9 (36%) had previous experience of PR. The duration of interviews ranged from 15 to 40 min. Three themes were generated from interview data: (1) facilitators and barriers to HBPR, (2) perceived changes from taking part in HBPR, and (3) how to improve HBPR in the future. Illustrative quotes are presented in Table 2. There were no notable differences in the views and experiences between those who had or had not previously participated in a PR programme.

**Table 2.** Qualitative themes and illustrative quotes.

Themes	Quotes
1. Facilitators and barriers to HBPR	“There is lot of advantage in this home-based rehab: 1. You don’t need to spend anything, 2. You don’t need to travel, 3. If you have equipment, you can do all the exercise” (P01) “I am happy with this program as I can continue my exercise program at home. You know I have remembered most of the exercises now, I am doing few exercises while watching TV and it’s easy for me to manage my baby and exercise at home which was tough in centre” (P19) “There are benefits for all with this program. Me, my son and my daughter in law all were doing exercises and we are getting benefit from our exercises. I can say this exercise is for everyone, anyone can do this and get benefits out of it. The most important thing is, it will give you mental as well as physical strength to everyone” (P27) “I think wife and kids are always encouraging me and they also used to remind me about my exercise. Sometimes, if I am getting late then my wife used to ask me and remind me about my exercise” (P06) “Sometimes when I have to skip my exercise due to visitors at home because I find it difficult to manage time” (P08) “Yes, there will be different challenges for different people like time management, visitors, relatives, household work but for me it was laziness and my health issues which I have told you” (P16) “Sometimes it was after work exertion, sometimes I must go for outing with family and children and sometimes it’s about mood” (P08) “I think at home we are doing exercise without supervision and according to our comfort and suitable situation. Sometimes it happened that I started exercising and after few minutes I stopped because of laziness” (P22) “Doing exercise at my place provides me flexibility of time but yes there is no strictness and it is difficult to get into regular schedule without focus and strictness” (P11)
2. Perceived changes from taking part in HBPR	“Yes, I have increased the numbers, in the beginning I was doing 3 sets of 10 reps for weight exercises now I am doing 15 into 3 and I have started with 15 min walking now I am doing 30–40 min” (P09) “In daily activities my difficulties during bathing and changing dress had been reduced a lot and other activities in which walking is involved it also become easier for me” (P04) “I was also using oxygen during walk, bathroom activity and during sleep but as I have started this program, I don’t have to use oxygen while bathroom activities or during sleep” (P23) “Earlier I was afraid to go out alone now if someone books a cab for me, I can travel alone so that is one thing which was not possible without exercise, basically I gained confidence” (P01) “They [other individuals with ILD] should go through the book [manual] as such. It let us know everything about our condition then they do things accordingly” (P01) “I would say, do not lose your hope. Follow the advice of your doctor and do regular exercises. It will make difference in your life and you can live a good life” (P03)
3. How to improve HBPR in the future	“Yes, just have one suggestion that if it would be Hindi version also then many common people of India who doesn’t know English can be benefitted by it” (P04) “I personally think you should suggest people to come to rehabilitation centre with book [manual], let them do exercises under you supervision for few days and then ask them to do it at home. It might be like 2 days at centre and rest at the days at home with book” (P20)

### Theme 1 - Facilitators and barriers to HBPR

The main advantage of HBPR described by participants was that it overcame the barriers related to centre-based PR. There were no time restrictions to do exercise at home, and reduced the stress burden of travel. Participants described family members actively supporting their exercise programme by exercising together, reading instructions from the manual, and correcting them as needed.

Some individuals faced challenges in completing the HBPR programme. Due to certain domestic circumstances, such as guest arrival or household work, it was difficult to find the time and follow a strict routine at home. Time flexibility and lack of supervision were among the reasons for reduced discipline and punctuality; therefore, some participants could not adhere to the program as intended.

### Theme 2 - Perceived changes from taking part in HBPR

Participants were satisfied with the increase in walking duration and exercise repetitions, and reported improvements in their functional abilities after completing the program. Furthermore, participants felt that their clinical symptoms were reduced and their independency in daily activities, such as washing, dressing, walking, and travelling had been enhanced, which improved their mental wellbeing and mood.

Belief in the value of exercise and self-management for their IPF was reported, with some participants wanting to recommend this intervention to other individuals with the condition.

### Theme 3 - How to improve HBPR in the future

Some participants felt that with minor alterations the HBPR programme would be suitable for a broader patient group. For example, translating the manual into the national language (Hindi). A hybrid model of both centre-based and HBPR was also recommended, especially for those who require a greater level of supervision to build self-confidence, understand exercise techniques, and follow an exercise routine.

### Secondary outcomes

Mean values for pre- and post-intervention secondary outcomes, along with change scores, are shown in Table 3. Following HBPR, MCIDs were reached for MRC dyspnoea, total KBILD, HADS depression and anxiety scores, ISWT distance, and five-times sit-to-stand time. All secondary outcome results are presented in Supplemental material H.

**Table 3.** Pre- and post-intervention values, and change scores for secondary outcomes.

Outcome measures	Pre	Post	Mean change	MCID
Health status (*n* = 20)				
MRC dyspnoea score	3.4 (1.1)	2.1 (0.8)	−1.2 (0.8)	−1 point
KBILD transformed scores				
Breathlessness and	36.3 (23.0)	65.7 (17.9)	29.3 (22.3)	4.4 points
activities	77.1 (19.5)	93.8 (8.1)	16.7 (18.9)	9.8 points
Chest symptoms	53.0 (18.7)	78.7 (13.5)	25.6 (16.6)	5.4 points
Psychological	54.7 (16.5)	78.2 (12.0)	23.5 (15.5)	3.9 points
Total				
CAT score	18.1 (6.2)	8.0 (5.6)	−10.1 (6.4)	−2 points
CCQ scores				
Symptom	2.4 (1.0)	1.2 (0.9)	−1.1 (1.0)	
Functional	3.2 (1.3)	1.5 (1.1)	−1.7 (1.2)	
Mental	2.5 (1.6)	1.2 (1.2)	−1.3 (1.3)	
Total	2.7 (0.9)	1.3 (0.9)	−1.4 (0.9)	0.4 points
HADS depression score	7.7 (4.1)	4.0 (3.5)	−3.7 (3.2)	2 points
HADS anxiety score	8.2 (4.1)	4.6 (3.9)	−3.6 (3.4)	2 points
EQ-5D-5 L visual analogue score	54.6 (11.5)	73.6 (13.7)	19.0 (10.4)	7.0 points
Exercise capacity (*n* = 20)				
ISWT, best distance (m)	268.5 (116.0)	321.0 (116.4)	52.5 (34.6)	33.4-35.0 m
ESWT, time taken (s)	272.2 (122.9)	392.8 (87.2)	120.6 (72.6)	170-209 s
5 x sit-to-stand, time taken (s)	12.7 (2.8)	8.6 (2.1)	−4.1 (2.1)	1.7 s

MRC: medical research council; KBILD: king’s brief interstitial lung disease; CAT: COPD assessment test; CCQ: clinical COPD questionnaire; HADS: hospital anxiety and depression scale; EQ-5D-5 L: european quality of life 5-dimensions, ISWT: incremental shuttle walk test, ESWT: endurance shuttle walk tests.

## Discussion

### Summary of the main findings

The aim of this trial was to assess the feasibility and acceptability of HBPR, via a culturally-tailored manual, among individuals with IPF in Delhi, India. The recruitment to and follow-up of the trial exceeded the criteria of feasibility, indicating that it is feasible to deliver the intervention. HBPR was acceptable to the majority of participants, suggesting that this is an appropriate mode of PR in this population and setting. Compliant participants reported perceived benefits, and those who were unable to fully adhere suggested improvements to the programme, including regular follow-ups from healthcare workers. Secondary outcome measures including exercise capacity, HRQoL, and mood showed potentially clinically meaningful improvements, aligning with the views of participants during interviews.

### Interpretation of results

The 83% uptake of individuals with ILD observed in this trial was higher than those observed for traditional centre-based PR (∼63% of eligible) in this context and population.^41^ A trial investigating the feasibility of HBPR in Malaysia reported a higher uptake rate (93%) but a lower completion rate (37%) due to COVID-19 restrictions.^42^ The 6-week completion rate of 83% for our ‘SPACEforILD’ intervention mirrors the original 80% completion rate of ‘SPACEforCOPD’ trial in the UK,^15^ and is greater than the completion rate reported in the UK national PR audit for COPD (67%). Significantly higher completion rates following HBPR compared to centre-based PR have also been reported in Australia.^43^ The reported facilitators to our HBPR intervention, specifically low costs, minimal burden of travel, more flexibility regarding when to perform exercises, and family support, are consistent with literature from both high-^44–46^ and LMICs.^10,13,42,47^ These noted advantages of HBPR may help to overcome the barriers associated with more traditional, centre-based PR in LMICs.^48^ The findings outlined above emphasize the benefit of having a menu of PR options to facilitate a patient choice approach to enhance the uptake and completion of PR in India.

Many of the female participants in this trial were not in work and identified as full-time homemakers, reflecting cultural norms in India, where women are primarily responsible for household duties. Such gendered expectations can limit women’s autonomy and confidence, often making them hesitant to travel alone, even for essential healthcare services like centre-based PR. Our HBPR intervention enabled women to care for their health without neglecting domestic responsibilities, which likely enhanced both its acceptability and adherence among these participants.

Participants in the present trial were provided with contact details for further support if needed, after the initial follow-up call. However, individuals reported the lack of direct supervision as the main barrier towards adhering to the programme. A previous trial investigating the acceptability of HBPR for individuals with COPD in Australia incorporated a home visit before weekly telephone calls to provide support, motivation, and education to participants.^46^ This was reported to be a facilitator to the predominantly unsupervised programme for the majority of individuals, with one participant suggesting the need for more direct supervision.^46^

Albeit not powered to detect significant improvements, we found promising changes in clinical outcomes, including dyspnoea, knowledge of ILD, anxiety, depression, exercise capacity, and lower limb physical function. A randomised controlled trial (RCT) assessing the effect of the ‘SPACEforCOPD’ manual found significant improvements in dyspnoea,^15^ exercise capacity, and anxiety compared to usual care following the 6-weeks intervention comprising exercise and self-management strategies. Furthermore, ‘SPACEforCOPD’ is a cost-effective intervention within the UK-based healthcare system.^49^ In India, centre-based PR for individuals with ILD has been shown to improve HRQoL and exercise capacity.^41,50,51^ A recent trial conducted in Manipal, India found similar improvements in these outcomes following a 4-weeks HBPR intervention.^16^ Together, these findings demonstrate the potential for exercise and improved self-management through HBPR to increase PR provision whilst maintaining a high quality of service and patient care in India.

### Strengths and limitations

This trial utilised a qualitative and quantitative approach to assess the feasibility and acceptability of a contextually adapted version of the evidence-based ‘SPACEforCOPD’ manual for individuals living with IPF in Delhi, India. The development of our manual involved individuals with IPF who provided important modifications and suggestions regarding cultural adaptations. The qualitative aspect of this trial allowed us to determine the acceptability of the intervention, and identify various facilitators, barriers, and future improvements to inform/improve future HBPR trials.

This was a single-centre trial conducted in Delhi, and therefore, findings are unlikely to be generalisable to India as a whole due to its large population and various cultures across different regions. According to the India Human Development Survey (IHDS), about 20% of Indian adults have some ability to speak English, with 4% reporting that they are fluent, while another 16% report having limited conversational skills.^52^ English language access is also associated with urbanization, and India has a low urban ratio to rural settings,^53^ thus, Hindi as the national language, is the most widely spoken across the country. Our trial participants were an affluent and highly educated population with the majority able to read and understand English but as well as not being in work. The reported suggestion for a more hybrid approach may reflect their employment status (70% not in employment) allowing them greater possibility to attend supervised centre-based PR sessions. The ‘SPACEforILD’ manual will be translated into Hindi and other local languages to facilitate implementation and testing in other areas and populations across India.

Due to the single-arm design of the trial, the interpretation of clinical results is limited, and details regarding exercise adherence were gathered subjectively, via interviews, only. For the original ‘SPACEforCOPD’ manual, between-group changes were only maintained for the ESWT at 6-months follow-up.^15^ The long-term follow-up of ‘SPACEforILD’ remains unclear, emphasizing the need for a fully-powered RCT in this population.

### Future recommendations

The finding that contextually-adapted HBPR via the ‘SPACEforILD’ manual is both feasible and acceptable from this trial justifies the need for a fully-powered RCT to assess the clinical- and cost-effectiveness of this intervention. Future work will need to address the lack of validated outcome measures in this population. Qualitative findings from the present trial emphasize the importance of implementing ‘SPACEforILD’ within a ‘patient choice’ model of PR, e.g., alongside secondary care and community options. Furthermore, there is a need to develop this manual in multiple languages to reach a broader range of individuals across India and beyond, into other LMICs and settings where digital PR options may not be suitable or easily accessible. Future work should aim to assess the feasibility of manual/HBPR for those with other chronic respiratory diseases, e.g., post-tuberculosis lung disease, and those with multiple long-term conditions.

## Conclusion

Culturally-tailored, manual-based HBPR is feasible and acceptable in individuals with IPF in Delhi, India. Facilitators included less time restrictions to exercise, reduced need to travel, and family support. The main barrier reported was the lack of supervision resulting in reduced adherence, and recommendations suggested by participants included the need to translate the manual into a range of languages, and to implement it into a more hybrid approach. A fully-powered RCT is needed to assess the clinical- and cost-effectiveness of this intervention.

## Supplemental Material

Supplemental Material - Feasibility and acceptability of home-based pulmonary rehabilitation for individuals with idiopathic pulmonary fibrosis in Delhi, India Supplemental Material for Feasibility and acceptability of home-based pulmonary rehabilitation for individuals with idiopathic pulmonary fibrosis in Delhi, India by Humaira Hanif, Obaidullah Ahmed, James Manifield, Mohd Shibli, Amy Barradell, Zahira Ahmed, Dominic Malcolm, Andy Barton, Deepak Talwar, Mark W Orme and Sally J Singh in Chronic Respiratory Disease

## References

[1] Maher TM Bendstrup E Dron L , et al. Global incidence and prevalence of idiopathic pulmonary fibrosis. Respir Res 2021; 22: 197–210. 34233665 10.1186/s12931-021-01791-z PMC8261998

[2] Dhooria S Sehgal IS Agarwal R , et al. Incidence, prevalence, and national burden of interstitial lung diseases in India: estimates from two studies of 3089 subjects. PLoS One 2022; 17: e0271665. 35862355 10.1371/journal.pone.0271665 PMC9302724

[3] Raghu G Collard HR Egan JJ , et al. An official ATS/ERS/JRS/ALAT statement: idiopathic pulmonary fibrosis: evidence-based guidelines for diagnosis and management. Am J Respir Crit Care Med 2011; 183: 788–824. 21471066 10.1164/rccm.2009-040GL PMC5450933

[4] Raghu G Remy-Jardin M Richeldi L , et al. Idiopathic pulmonary fibrosis (an update) and progressive pulmonary fibrosis in adults: an official ATS/ERS/JRS/ALAT clinical practice guideline. Am J Respir Crit Care Med 2022; 205: e18–e47. 35486072 10.1164/rccm.202202-0399ST PMC9851481

[5] Collard HR Ward AJ Lanes S , et al. Burden of illness in idiopathic pulmonary fibrosis. J Med Econ 2012; 15: 829–835. 22455577 10.3111/13696998.2012.680553

[6] Cox IA Arriagada NB De Graaff B , et al. Health-related quality of life of patients with idiopathic pulmonary fibrosis: a systematic review and meta-analysis. Eur Respir Rev 2020; 29(158): 200154. 33153990 10.1183/16000617.0154-2020 PMC9488638

[7] Swigris J Kuschner W Jacobs S , et al. Health-related quality of life in patients with idiopathic pulmonary fibrosis: a systematic review. Thorax 2005; 60: 588–594. 15994268 10.1136/thx.2004.035220 PMC1747452

[8] Rochester CL Alison JA Carlin B , et al. Pulmonary rehabilitation for adults with chronic respiratory disease: an official American thoracic society clinical practice guideline. Am J Respir Crit Care Med 2023; 208: e7–e26. 37581410 10.1164/rccm.202306-1066ST PMC10449064

[9] Singh SJ Halpin DM Salvi S , et al. Exercise and pulmonary rehabilitation for people with chronic lung disease in LMICs: challenges and opportunities. Lancet Respir Med 2019; 7: 1002–1004. 31629670 10.1016/S2213-2600(19)30364-9

[10] Hanif H Ahmed O Manifield J , et al. Understanding the lived experience of idiopathic pulmonary fibrosis and how this shapes views on home-based pulmonary rehabilitation in Delhi, India. Chron Respir Dis 2024; 21: 14799731241258216. 38787595 10.1177/14799731241258216 PMC11127573

[11] Thakor M Singh V Manifield J , et al. Community-based pulmonary rehabilitation in an economically deprived area of Jodhpur, India: a mixed-methods feasibility trial. Int J Chronic Obstr Pulm Dis 2025; 20: 473–478. 10.2147/COPD.S488766 PMC11878114 40041473

[12] Augustine A Bhat A Vaishali K , et al. Barriers to pulmonary rehabilitation–A narrative review and perspectives from a few stakeholders. Lung India 2021; 38: 59–63. 33402639 10.4103/lungindia.lungindia_116_20 PMC8066922

[13] Padhye R Sahasrabudhe SD Orme MW , et al. Perspectives of patients with chronic respiratory diseases and medical professionals on pulmonary rehabilitation in Pune, India: qualitative analysis. JMIR Form Res 2023; 7: e45624. 37934558 10.2196/45624 PMC10664007

[14] Apps LD Mitchell KE Harrison SL , et al. The development and pilot testing of the self-management programme of activity, coping and education for chronic obstructive pulmonary disease (SPACE for COPD). Int J Chronic Obstr Pulm Dis 2013; 8: 317–327. 10.2147/COPD.S40414 PMC3711650 23874093

[15] Mitchell KE Johnson-Warrington V Apps LD , et al. A self-management programme for COPD: a randomised controlled trial. Eur Respir J 2014; 44: 1538–1547. 25186259 10.1183/09031936.00047814

[16] Amin R Vaishali K Maiya GA , et al. Influence of home-based pulmonary rehabilitation program among people with interstitial lung disease: a pre-post study. Physiother Theory Pract 2023; 40: 2265–2273. 37603451 10.1080/09593985.2023.2245878

[17] Bolton CE Bevan-Smith EF Blakey JD , et al. British thoracic society guideline on pulmonary rehabilitation in adults: accredited by NICE. Thorax 2013; 68: ii1–ii30. 23880483 10.1136/thoraxjnl-2013-203808

[18] Orme MW Free RC Manise A , et al. Global RECHARGE: establishing a standard international data set for pulmonary rehabilitation in low-and middle-income countries. J Glob Health 2020; 10: 020316. 33282213 10.7189/jogh.10.020316 PMC7688060

[19] Eldridge SM Chan CL Campbell MJ , et al. CONSORT 2010 statement: extension to randomised pilot and feasibility trials. BMJ 2016; 355: i5239. 27777223 10.1136/bmj.i5239 PMC5076380

[20] Hoffmann TC Glasziou PP Boutron I , et al. Better reporting of interventions: template for intervention description and replication (TIDieR) checklist and guide. BMJ 2014; 348: g1687. 24609605 10.1136/bmj.g1687

[21] Tong A Sainsbury P Craig J . Consolidated criteria for reporting qualitative research (COREQ): a 32-item checklist for interviews and focus groups. Int J Qual Health Care 2007; 19: 349–357. 17872937 10.1093/intqhc/mzm042

[22] Raghu G Remy-Jardin M Myers JL , et al. Diagnosis of idiopathic pulmonary fibrosis. An official ATS/ERS/JRS/ALAT clinical practice guideline. Am J Respir Crit Care Med 2018; 198: e44–e68. 30168753 10.1164/rccm.201807-1255ST

[23] Wok Zaki A Darmi R . CEFR: education towards 21st century of learning. Why matters. J Soc Sci Humanit 2021; 4: 14–20.

[24] Totton N Lin J Julious S , et al. A review of sample sizes for UK pilot and feasibility studies on the ISRCTN registry from 2013 to 2020. Pilot Feasibility Stud 2023; 9: 188. 37990337 10.1186/s40814-023-01416-w PMC10662929

[25] Whitehead AL Julious SA Cooper CL , et al. Estimating the sample size for a pilot randomised trial to minimise the overall trial sample size for the external pilot and main trial for a continuous outcome variable. Stat Methods Med Res 2016; 25: 1057–1073. 26092476 10.1177/0962280215588241 PMC4876429

[26] Holland AE Cox NS Houchen-Wolloff L , et al. Defining modern pulmonary rehabilitation. An official American thoracic society workshop report. Ann Am Thorac Soc 2021; 18: e12–e29. 33929307 10.1513/AnnalsATS.202102-146ST PMC8086532

[27] Man W Chaplin E Daynes E , et al. British thoracic society clinical statement on pulmonary rehabilitation. Thorax 2023; 78: s2–s15. 37770084 10.1136/thorax-2023-220439

[28] Harris PA Taylor R Minor BL , et al. The REDCap consortium: building an international community of software platform partners. J Biomed Inf 2019; 95: 103208. 10.1016/j.jbi.2019.103208 PMC7254481 31078660

[29] Harris PA Taylor R Thielke R , et al. Research electronic data capture (REDCap)—a metadata-driven methodology and workflow process for providing translational research informatics support. J Biomed Inf 2009; 42: 377–381. 10.1016/j.jbi.2008.08.010 PMC2700030 18929686

[30] Group TE . EuroQol-a new facility for the measurement of health-related quality of life. Health Policy 1990; 16: 199–208. 10109801 10.1016/0168-8510(90)90421-9

[31] Patel AS Siegert RJ Brignall K , et al. The development and validation of the king's brief interstitial lung disease (K-BILD) health status questionnaire. Thorax 2012; 67: 804–810. 22555278 10.1136/thoraxjnl-2012-201581

[32] Stenton C . The MRC breathlessness scale. Occup Med 2008; 58: 226–227. 10.1093/occmed/kqm162 18441368

[33] Jones P Harding G Berry P , et al. Development and first validation of the COPD assessment test. Eur Respir J 2009; 34: 648–654. 19720809 10.1183/09031936.00102509

[34] Van der Molen T Willemse BW Schokker S , et al. Development, validity and responsiveness of the clinical COPD questionnaire. Health Qual Life Outcome 2003; 1: 13. 10.1186/1477-7525-1-13 PMC156640 12773199

[35] Zigmond AS Snaith RP . The hospital anxiety and depression scale. Acta Psychiatr Scand 1983; 67: 361–370. 6880820 10.1111/j.1600-0447.1983.tb09716.x

[36] Revill S Morgan M Singh S , et al. The endurance shuttle walk: a new field test for the assessment of endurance capacity in chronic obstructive pulmonary disease. Thorax 1999; 54: 213–222. 10325896 10.1136/thx.54.3.213 PMC1745445

[37] Singh SJ Morgan M Scott S , et al. Development of a shuttle walking test of disability in patients with chronic airways obstruction. Thorax 1992; 47: 1019–1024. 1494764 10.1136/thx.47.12.1019 PMC1021093

[38] Bohannon RW . Sit-to-stand test for measuring performance of lower extremity muscles. Percept Mot Skills 1995; 80: 163–166. 7624188 10.2466/pms.1995.80.1.163

[39] Brooks J McCluskey S Turley E , et al. The utility of template analysis in qualitative psychology research. Qual Res Psychol 2015; 12: 202–222. 27499705 10.1080/14780887.2014.955224 PMC4960514

[40] Braun V Clarke V . One size fits all? What counts as quality practice in (reflexive) thematic analysis? Qual Res Psychol 2021; 18: 328–352.

[41] Sharma R Kaushal M Ali M , et al. Effect of respiratory muscle training and pulmonary rehabilitation on exercise capacity in patients with interstitial lung disease: a prospective quasi-experimental study. Eurasian J Pulmonol 2019; 21: 87.

[42] Chan SC Engksan JP Nathan JJ , et al. Developing a home-based pulmonary rehabilitation programme for patients with chronic respiratory diseases in Malaysia: a mixed-method feasibility study. J Glob Health 2023; 13: 04099. 37883199 10.7189/jogh.13.04099 PMC10602205

[43] Holland AE Mahal A Hill CJ , et al. Home-based rehabilitation for COPD using minimal resources: a randomised, controlled equivalence trial. Thorax 2017; 72: 57–65. 27672116 10.1136/thoraxjnl-2016-208514 PMC5329049

[44] Apps LD Harrison SL Mitchell KE , et al. A qualitative study of patients’ experiences of participating in SPACE for COPD: a self-management programme of activity, coping and education. ERJ Open Res 2017; 3: 00017–02017. 29204434 10.1183/23120541.00017-2017 PMC5703355

[45] Houchen-Wolloff L Orme M Barradell A , et al. Web-based self-management program (SPACE for COPD) for individuals hospitalized with an acute exacerbation of chronic obstructive pulmonary disease: nonrandomized feasibility trial of acceptability. JMIR Mhealth Uhealth 2021; 9: e21728. 34114960 10.2196/21728 PMC8235284

[46] Lahham A McDonald CF Mahal A , et al. Home-based pulmonary rehabilitation for people with COPD: a qualitative study reporting the patient perspective. Chron Respir Dis 2018; 15: 123–130. 28868892 10.1177/1479972317729050 PMC5958468

[47] Singh DN Kaur H Roy S , et al. Needs assessment for introducing pulmonary rehabilitation for chronic obstructive pulmonary disease management in a rural Indian setting: a qualitative study. BMJ Open Respir Res 2023; 10: e001696. 10.1136/bmjresp-2023-001696 PMC10360411 37474198

[48] Bickton FM Shannon H . Barriers and enablers to pulmonary rehabilitation in low-and middle-income countries: a qualitative study of healthcare professionals. Int J Chronic Obstr Pulm Dis 2022; 17: 141–153. 10.2147/COPD.S348663 PMC8763198 35046649

[49] Dritsaki M Johnson-Warrington V Mitchell K , et al. An economic evaluation of a self-management programme of activity, coping and education for patients with chronic obstructive pulmonary disease. Chron Respir Dis 2016; 13: 48–56. 26703923 10.1177/1479972315619578 PMC5720203

[50] Gupta K Kumar R Gaur S . Effect of a domiciliary pulmonary rehabilitation programme on disability in patients with interstitial lung diseases. Indian J Chest Dis Allied Sci 2007; 49: 213. 16022153

[51] Vivek K Janmeja AK Aggarwal D , et al. Pulmonary rehabilitation in patients with interstitial lung diseases in an outpatient setting: a randomised controlled trial. Indian J Chest Dis Allied Sci 2017; 59: 75–80.

[52] Azam M Chin A Prakash N . The returns to english-language skills in India. Econ Dev Cult Change 2013; 61: 335–367.

[53] Parshad RD Bhowmick S Chand V , et al. What is India speaking? Exploring the “Hinglish” invasion. Phys Stat Mech Appl 2016; 449: 375–389.

