# Supplementary material for: Feasibility and acceptability of home-based pulmonary rehabilitation for individuals with idiopathic pulmonary fibrosis in Delhi, India
Source: Chron Respir Dis. 2025 Sep 3;22:14799731251375043. doi: 10.1177/14799731251375043 (PMC12409018; doi:10.1177/14799731251375043)
Supplement: Supplemental Material - Feasibility and acceptability of home-based pulmonary rehabilitation for individuals with idiopathic pulmonary fibrosis in Delhi, India [file sj-pdf-1-crd-10.1177_14799731251375043.pdf]

Supplementary material

Contents

Supplementary material A – CONSORT checklist 2
Supplementary material B – TIDieR checklist 5
Supplementary material C – COREQ checklist 8
Supplementary material D - SPACE for ILD manual 9
Supplementary material E – Interview topic guide 12
Supplementary material F – Secondary outcome measures 13
Supplementary material G - Baseline characteristics 15
Supplementary material H - Secondary outcome results 16

Supplementary material A – CONSORT checklist

CONSORT 2010 checklist of information to include when reporting a pilot or feasibility trial*

Section/Topic	Item No	Checklist item	Reported on page No
**Title and abstract**			
	1a	Identification as a pilot or feasibility randomised trial in the title	1
	1b	Structured summary of pilot trial design, methods, results, and conclusions (for specific guidance see CONSORT abstract extension for pilot trials)	2
**Introduction**			
Background and objectives	2a	Scientific background and explanation of rationale for future definitive trial, and reasons for randomised pilot trial	3
	2b	Specific objectives or research questions for pilot trial	4
**Methods**			
Trial design	3a	Description of pilot trial design (such as parallel, factorial) including allocation ratio	4
	3b	Important changes to methods after pilot trial commencement (such as eligibility criteria), with reasons	N/A
Participants	4a	Eligibility criteria for participants	5
	4b	Settings and locations where the data were collected	5
	4c	How participants were identified and consented	5
Interventions	5	The interventions for each group with sufficient details to allow replication, including how and when they were actually administered	5-7
Outcomes	6a	Completely defined prespecified assessments or measurements to address each pilot trial objective specified in 2b, including how and when they were assessed	7
	6b	Any changes to pilot trial assessments or measurements after the pilot trial commenced, with reasons	N/A
	6c	If applicable, prespecified criteria used to judge whether, or how, to proceed with future definitive trial	7

Sample size	7a	Rationale for numbers in the pilot trial	5
	7b	When applicable, explanation of any interim analyses and stopping guidelines	N/A
Randomisation:			
Sequence generation	8a	Method used to generate the random allocation sequence	N/A
	8b	Type of randomisation(s); details of any restriction (such as blocking and block size)	N/A
Allocation concealment mechanism	9	Mechanism used to implement the random allocation sequence (such as sequentially numbered containers), describing any steps taken to conceal the sequence until interventions were assigned	N/A
Implementation	10	Who generated the random allocation sequence, who enrolled participants, and who assigned participants to interventions	N/A
Blinding	11a	If done, who was blinded after assignment to interventions (for example, participants, care providers, those assessing outcomes) and how	N/A
	11b	If relevant, description of the similarity of interventions	N/A
Statistical methods	12	Methods used to address each pilot trial objective whether qualitative or quantitative	8-9
**Results**			
Participant flow (a diagram is strongly recommended)	13a	For each group, the numbers of participants who were approached and/or assessed for eligibility, randomly assigned, received intended treatment, and were assessed for each objective	9/Fig 1.
	13b	For each group, losses and exclusions after randomisation, together with reasons	9/Fig 1.
Recruitment	14a	Dates defining the periods of recruitment and follow-up	5
	14b	Why the pilot trial ended or was stopped	N/A
Baseline data	15	A table showing baseline demographic and clinical characteristics for each group	10/Table 1
Numbers analysed	16	For each objective, number of participants (denominator) included in each analysis. If relevant, these numbers should be by randomised group	9-11
Outcomes and estimation	17	For each objective, results including expressions of uncertainty (such as 95% confidence interval) for any estimates. If relevant, these results should be by randomised group	N/A

Ancillary analyses	18	Results of any other analyses performed that could be used to inform the future definitive trial	11-15/Table 2 and 3
Harms	19	All important harms or unintended effects in each group (for specific guidance see CONSORT for harms)	N/A
	19a	If relevant, other important unintended consequences	N/A
**Discussion**			
Limitations	20	Pilot trial limitations, addressing sources of potential bias and remaining uncertainty about feasibility	17-18
Generalisability	21	Generalisability (applicability) of pilot trial methods and findings to future definitive trial and other studies	17
Interpretation	22	Interpretation consistent with pilot trial objectives and findings, balancing potential benefits and harms, and considering other relevant evidence	16-18
	22a	Implications for progression from pilot to future definitive trial, including any proposed amendments	18
**Other information**			
Registration	23	Registration number for pilot trial and name of trial registry	2/4
Protocol	24	Where the pilot trial protocol can be accessed, if available	4
Funding	25	Sources of funding and other support (such as supply of drugs), role of funders	2
	26	Ethical approval or approval by research review committee, confirmed with reference number	4

Citation: Eldridge SM, Chan CL, Campbell MJ, Bond CM, Hopewell S, Thabane L, et al. CONSORT 2010 statement: extension to randomised pilot and feasibility trials. BMJ. 2016;355. This is an Open Access article distributed in accordance with the terms of the Creative Commons Attribution (CC BY 3.0) license (http://creativecommons.org/licenses/by/3.0/), which permits others to distribute, remix, adapt and build upon this work, for commercial use, provided the original work is properly cited.

*We strongly recommend reading this statement in conjunction with the CONSORT 2010, extension to randomised pilot and feasibility trials, Explanation and Elaboration for important clarifications on all the items. If relevant, we also recommend reading CONSORT extensions for cluster randomised trials, non-inferiority and equivalence trials, non-pharmacological treatments, herbal interventions, and pragmatic trials. Additional extensions are forthcoming: for those and for up-to-date references relevant to this checklist, see www.consort-statement.org.

Supplementary material B – TIDieR checklist

TIDieR
Template for Intervention Description and Replication

The TIDieR (Template for Intervention Description and Replication) Checklist*:

Information to include when describing an intervention and the location of the information

Item number	Item	Where located ** Primary paper (page or appendix number)	Other † (details)
	**BRIEF NAME**		
**1.**	Provide the name or a phrase that describes the intervention.	______5______	_______________
	**WHY**		
**2.**	Describe any rationale, theory, or goal of the elements essential to the intervention.	_____3-4____	______________
	**WHAT**		
**3.**	Materials: Describe any physical or informational materials used in the intervention, including those provided to participants or used in intervention delivery or in training of intervention providers. Provide information on where the materials can be accessed (e.g. online appendix, URL).	_____5-6____	______________
**4.**	Procedures: Describe each of the procedures, activities, and/or processes used in the intervention, including any enabling or support activities.	______6_____	______________
	**WHO PROVIDED**		
**5.**	For each category of intervention provider (e.g. psychologist, nursing assistant), describe their expertise, background and any specific training given.	______5-7____	______________

	**HOW**		
**6.**	Describe the modes of delivery (e.g. face-to-face or by some other mechanism, such as internet or telephone) of the intervention and whether it was provided individually or in a group.	______5/6____	______________
	**WHERE**		
**7.**	Describe the type(s) of location(s) where the intervention occurred, including any necessary infrastructure or relevant features.	______5/6____	______________
	**WHEN and HOW MUCH**		
**8.**	Describe the number of times the intervention was delivered and over what period of time including the number of sessions, their schedule, and their duration, intensity or dose.	______6______	______________
	**TAILORING**		
**9.**	If the intervention was planned to be personalised, titrated or adapted, then describe what, why, when, and how.	______6______	______________
	**MODIFICATIONS**		
**10.**‡	If the intervention was modified during the course of the study, describe the changes (what, why, when, and how).	______N/A____	______________
	**HOW WELL**		
**11.**	Planned: If intervention adherence or fidelity was assessed, describe how and by whom, and if any strategies were used to maintain or improve fidelity, describe them.	______N/A____	______________
**12.**‡	Actual: If intervention adherence or fidelity was assessed, describe the extent to which the intervention was delivered as planned.	______N/A_____	______________

** **Authors** - use N/A if an item is not applicable for the intervention being described. **Reviewers** – use '?' if information about the element is not reported/not sufficiently reported.

† If the information is not provided in the primary paper, give details of where this information is available. This may include locations such as a published protocol or other published papers (provide citation details) or a website (provide the URL).

ǂ If completing the TIDieR checklist for a protocol, these items are not relevant to the protocol and cannot be described until the study is complete.

* We strongly recommend using this checklist in conjunction with the TIDieR guide (see *BMJ* 2014;348:g1687) which contains an explanation and elaboration for each item.

* The focus of TIDieR is on reporting details of the intervention elements (and where relevant, comparison elements) of a study. Other elements and methodological features of studies are covered by other reporting statements and checklists and have not been duplicated as part of the TIDieR checklist. When a **randomised trial** is being reported, the TIDieR checklist should be used in conjunction with the CONSORT statement (see www.consort-statement.org) as an extension of **Item 5 of the CONSORT 2010 Statement.** When a **clinical trial protocol** is being reported, the TIDieR checklist should be used in conjunction with the SPIRIT statement as an extension of **Item 11 of the SPIRIT 2013 Statement** (see www.spirit-statement.org). For alternate study designs, TIDieR can be used in conjunction with the appropriate checklist for that study design (see www.equator-network.org).

Supplementary material C – COREQ checklist

Topic	Item No.	Guide Questions/Description	Reported on Page No.
**Domain 1: Research team and reflexivity**			
Personal characteristics			
Interviewer/facilitator	1	Which author/s conducted the interview or focus group?	7
Credentials	2	What were the researcher's credentials? E.g. PhD, MD	7
Occupation	3	What was their occupation at the time of the study?	7
Gender	4	Was the researcher male or female?	7
Experience and training	5	What experience or training did the researcher have?	7
Relationship with participants			
Relationship established	6	Was a relationship established prior to study commencement?	7-8
Participant knowledge of the interviewer	7	What did the participants know about the researcher? e.g. personal goals, reasons for doing the research	7
Interviewer characteristics	8	What characteristics were reported about the interviewer/facilitator? e.g. Bias, assumptions, reasons and interests in the research topic	87
**Domain 2: Study design**			
Theoretical framework			
Methodological orientation and Theory	9	What methodological orientation was stated to underpin the study? e.g. grounded theory, discourse analysis, ethnography, phenomenology, content analysis	8
Participant selection			
Sampling	10	How were participants selected? e.g. purposive, convenience, consecutive, snowball	5
Method of approach	11	How were participants approached? e.g. face-to-face, telephone, mail, email	5
Sample size	12	How many participants were in the study?	9/11
Non-participation	13	How many people refused to participate or dropped out? Reasons?	9/10
Setting			
Setting of data collection	14	Where was the data collected? e.g. home, clinic, workplace	11
Presence of non-participants	15	Was anyone else present besides the participants and researchers?	N/A
Description of sample	16	What are the important characteristics of the sample? e.g. demographic data, date	Table 1
Data collection			
Interview guide	17	Were questions, prompts, guides provided by the authors? Was it pilot tested?	Suppl E
Repeat interviews	18	Were repeat inter views carried out? If yes, how many?	N/A
Audio/visual recording	19	Did the research use audio or visual recording to collect the data?	8
Field notes	20	Were field notes made during and/or after the interview or focus group?	8
Duration	21	What was the duration of the inter views or focus group?	11
Data saturation	22	Was data saturation discussed?	N/A
Transcripts returned	23	Were transcripts returned to participants for comment and/or	N/A

Supplementary material D - SPACE for ILD manual

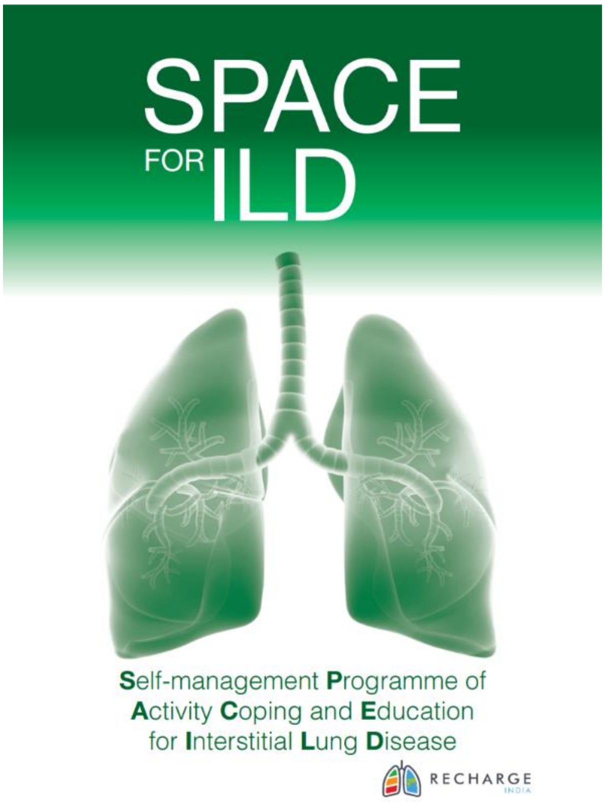

Content

Stage 1

What's happened to your lungs? 6
How to get fitter 9
Setting your goals 27
Managing your stress 30
Your emotions 32
Controlling your breathing 35
Fatigue and Management 44
Your medication 46

Stage 2

How to stay fit 52
Avoiding and managing days when you feel unwell 58
Saving your energy 64
The right foods when you feel unwell 69
Clearing your chest 73

Stage 3

How to get stronger 78
Managing your stress 86
Healthy eating 96
Travelling and your lung disease 109

Stage 4

Your hobbies and staying fit 114
Your relationships 123
Dealing with setbacks 126
Life- Foundation 129

Frequently Asked Questions & Appendix

Frequently asked questions 132
Setting your walking speed 135
Help for carers 141
Advice about oxygen 145
Smoking: advice on giving up 147
Information about your medication 152
Spare walking diaries 156
Spare strength training diary 157

Figure S1. The SPACE for ILD manual and its contents page.

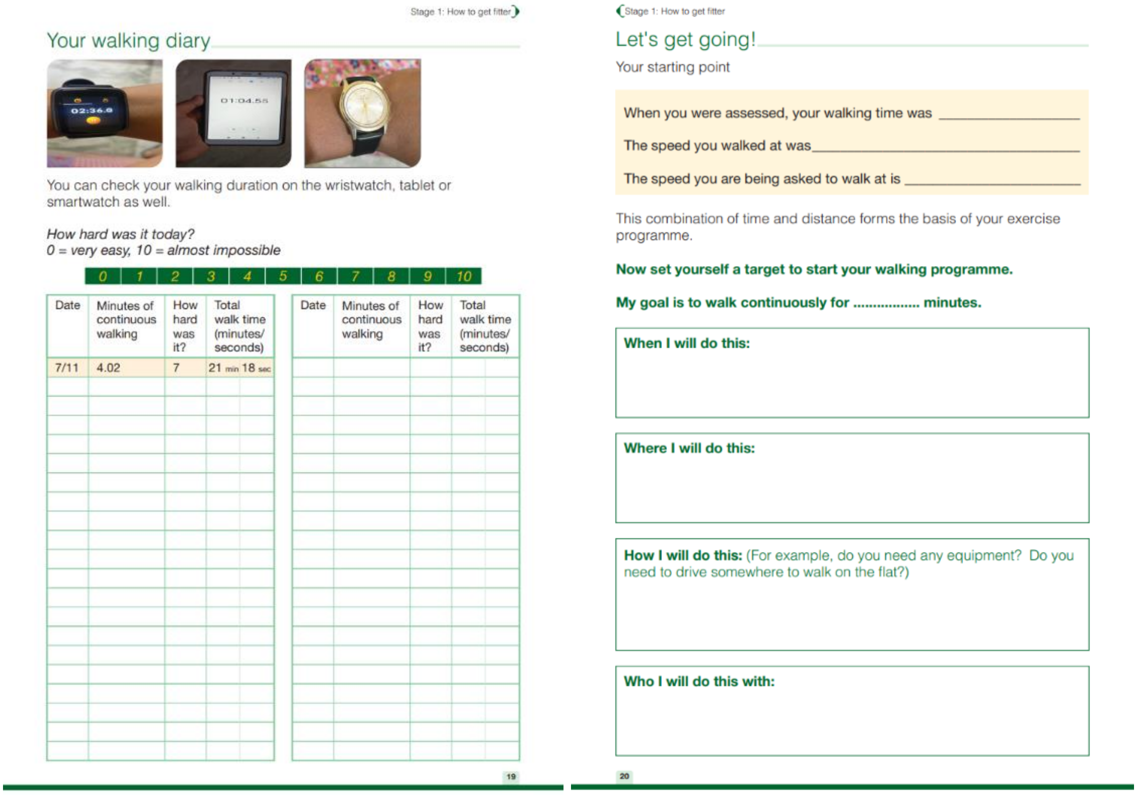

Stage 1: How to get fitter

Your walking diary

You can check your walking duration on the wristwatch, tablet or smartwatch as well.

How hard was it today?
0 = very easy, 10 = almost impossible

0	1	2	3	4	5	6	7	8	9	10

Date	Minutes of continuous walking	How hard was it?	Total walk time (minutes/ seconds)	Date	Minutes of continuous walking	How hard was it?	Total walk time (minutes/ seconds)
7/11	4.02	7	21 min 18 sec				

19

Stage 1: How to get fitter

Let's get going!

Your starting point

When you were assessed, your walking time was ________________

The speed you walked at was________________________________

The speed you are being asked to walk at is ____________________

This combination of time and distance forms the basis of your exercise programme.

Now set yourself a target to start your walking programme.

My goal is to walk continuously for .... minutes.

When I will do this:

Where I will do this:

How I will do this: (For example, do you need any equipment? Do you need to drive somewhere to walk on the flat?)

Who I will do this with:

20

Figure S2. Walking diary included in the SPACE for ILD manual.

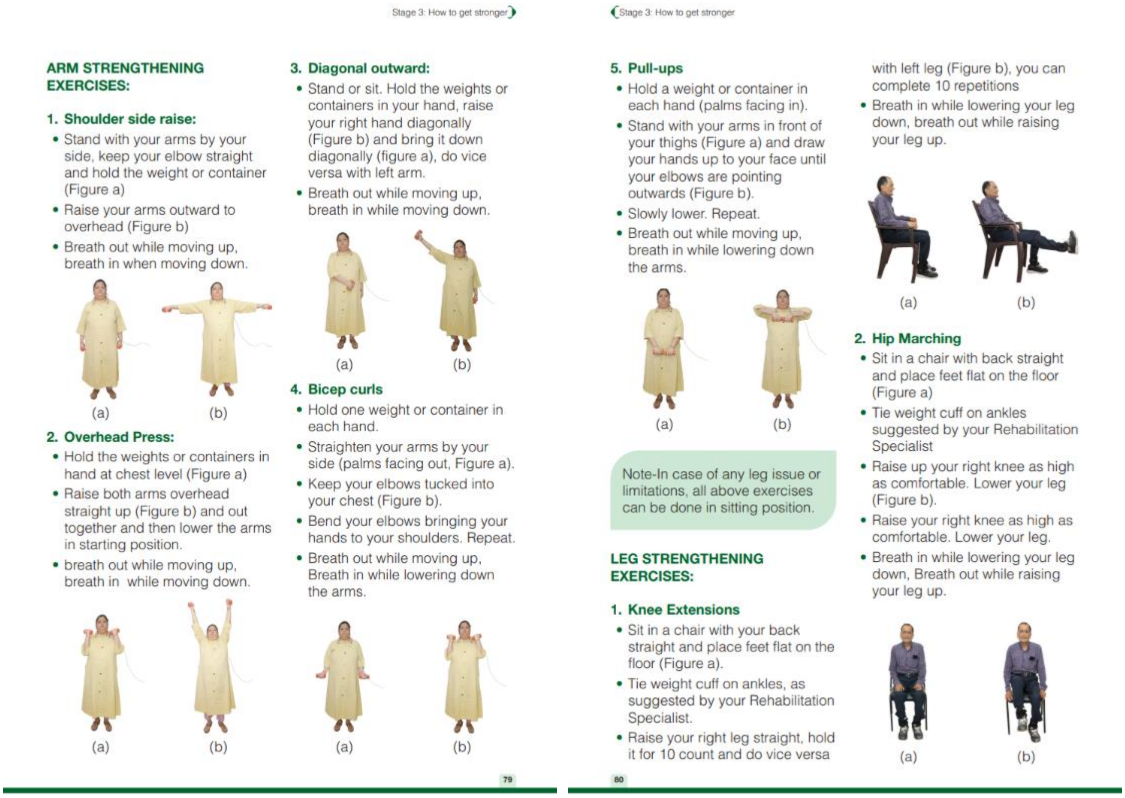

Stage 3: How to get stronger

ARM STRENGTHENING EXERCISES:

1. Shoulder side raise:
- Stand with your arms by your side, keep your elbow straight and hold the weight or container (Figure a)
- Raise your arms outward to overhead (Figure b)
- Breath out while moving up, breath in when moving down.

(a) (b)

2. Overhead Press:
- Hold the weights or containers in hand at chest level (Figure a)
- Raise both arms overhead straight up (Figure b) and out together and then lower the arms in starting position.
- breath out while moving up, breath in while moving down.

(a) (b)

3. Diagonal outward:
- Stand or sit. Hold the weights or containers in your hand, raise your right hand diagonally (Figure b) and bring it down diagonally (figure a), do vice versa with left arm.
- Breath out while moving up, breath in while moving down.

(a) (b)

4. Bicep curls
- Hold one weight or container in each hand.
- Straighten your arms by your side (palms facing out, Figure a).
- Keep your elbows tucked into your chest (Figure b).
- Bend your elbows bringing your hands to your shoulders. Repeat.
- Breath out while moving up, Breath in while lowering down the arms.

(a) (b)

79

Stage 3: How to get stronger

5. Pull-ups
- Hold a weight or container in each hand (palms facing in).
- Stand with your arms in front of your thighs (Figure a) and draw your hands up to your face until your elbows are pointing outwards (Figure b).
- Slowly lower. Repeat.
- Breath out while moving up, breath in while lowering down the arms.

(a) (b)

Note-In case of any leg issue or limitations, all above exercises can be done in sitting position.

LEG STRENGTHENING EXERCISES:

1. Knee Extensions
- Sit in a chair with your back straight and place feet flat on the floor (Figure a).
- Tie weight cuff on ankles, as suggested by your Rehabilitation Specialist.
- Raise your right leg straight, hold it for 10 count and do vice versa with left leg (Figure b), you can complete 10 repetitions
- Breath in while lowering your leg down, breath out while raising your leg up.

(a) (b)

2. Hip Marching
- Sit in a chair with back straight and place feet flat on the floor (Figure a)
- Tie weight cuff on ankles suggested by your Rehabilitation Specialist
- Raise up your right knee as high as comfortable. Lower your leg (Figure b).
- Raise your right knee as high as comfortable. Lower your leg.
- Breath in while lowering your leg down, Breath out while raising your leg up.

(a) (b)

80

Figure S3. Examples of resistance exercises with the SPACE for ILD manual.

The SPACEforILD manual was divided into four stages. Stage 1 included information about ILD and how to get fitter, stage 2 included information on how to stay fit, stage 3 involved how to get stronger and stress management, and stage 4 included advice on dealing with setbacks and relationships (Figure S1).

Specific cultural adaptations, informed by patient and public involvement (PPI) and previous qualitative work,[1] included: 1) Case studies with relatable situations and goals were incorporated, 2) details on energy-saving techniques, dietary information, and food items were adapted to be relevant for the Indian population, 3) yoga breathing techniques were added as it is culturally relevant,[2] 4) travelling and medical insurance guidance were included, and 5) information about the Lung India Fibrosis Expert Foundation (LIFE) was added.

The exercise programme consisted of individually prescribed walking and resistance training of the upper and lower limbs. Participants were asked to walk at least thirty minutes daily with the option of splitting this time into more manageable bouts prescribed based on baseline shuttle walk tests. They were encouraged to gradually increase the length of walking bouts throughout the programme. Walking diaries were provided to monitor adherence and progression (Figure S2). Participants were advised to perform resistance exercises of both upper and lower muscle groups (Figure S3) 3 times/week and gradually progress the weight by 0.5 kg.

Supplementary material E – Interview topic guide

Interviews focused on the following topics:

- **Expectations of PR:** reasons for taking part in this study, knowledge of PR prior to taking part in this trial, expectations towards (HB)PR before starting.
- **Experiences of HBPR:** positive (What went well? What was enjoyable?) and negative (What did not go well? What was less enjoyable//difficult?). What did your family members think to the HBPR?
- **Home-based nature of the intervention:** advantages and disadvantages, preference in terms of mode of delivery.
- **Adherence:** frequency, duration, type, and progression of exercises. Were you performing exercises along or did you have assistance from anyone? Did you complete training diaries?
- **Challenges and barriers:** job/household work, manual content, family support, environmental factors.
- **Perceived changes following HBPR:** have you noticed any changes in symptoms, physical activity levels etc.
- **Recommendations for future PR programmes:** suggested improvements for designing future PR programmes, suggested changes to manual.

Supplementary material F – Secondary outcome measures

Health-related quality of life

The European Quality of Life 5-Dimensions (EQ-5D-5L) questionnaire was used to measure health-related quality of life across five dimensions: mobility, self-care, usual activities, pain or discomfort and anxiety or depression [3]. This questionnaire is responsive to change following pulmonary rehabilitation, and has a MCID of 0.05 (utility index) and 7.0 (visual analogue scale) [4].

The King's Brief Interstitial Lung Disease (KBILD) was used to measure disease-specific health-related quality of life. It comprises of 15 items with three domains (psychological, breathlessness and activities and chest symptoms) [5]. The KBILD has been shown to be valid and reproducible [5], as well as responsive following PR, with an MCID of 3.9 points for the total score [6].

The Clinical COPD questionnaire (CCQ) consists of 10 items, each scored between 0-6, and divided into three domains (symptoms, functional, mental) [7]. The total score is calculated averaging the individual item scores, with higher CCQ scores representing worse quality of life. The CCQ is responsive to PR with an estimated MCID of 0.4 points [8].

The COPD Assessment Test (CAT) consists of eight items, each scored between 0 and 5 scored with a range of 0 to 40; scores of 0-10, 11-20, 21-30, 31-40 representing mild, moderate, severe or very severe negative impact on health-related quality of life, respectively [9]. The CAT is responsive to the effects of PR with an estimated MCID of 2 points [10].

Exercise capacity

Incremental Shuttle Walk Test (ISWT)

Participants were required to walk 10-meter shuttles as described previously [11], with walking speeds externally paced by an audio signal. They were instructed to reach the opposite cone at the time of each "bleep" which increased in frequency each minute and to walk until they were unable to keep up with the walking pace. A triple bleep indicated to the participant to increase their walking speed by 0.17 m/s. The test was terminated if the participant was unable to reach the cone (>0.5 m away) before the bleep on two consecutive occasions. No encouragement was provided by the researchers during this test. Participants performed this test twice (separated by 30 minutes), with the first being a practice test. The ISWT has been shown to be a valid measure that is response to PR in individuals with IPF, and MCID estimates range from 33.4-35.0 m difference [12].

Reasons for termination of the test were recorded (i.e., shortness of breath, leg fatigue, SpO_2 <80%, and unable to keep up with the bleeps) along with immediately pre- and post-ISWT measures of

oxygen saturation (SpO_2), heart rate, Borg breathlessness, and ratings of perceived exertion (RPE) scores.

Endurance Shuttle Walk Test (ESWT)

This test involved participants walking 10-meter shuttles at a fixed speed which was paced by an audio signal corresponding to 85% of their peak ISWT speed, as described previously [13]. The test featured 16 distinct walking speeds dictated by audio cues. As for the ISWT, participants were instructed to walk until they were unable to keep up with the walking pace, and the test was terminated if they did not reach the cone (>0.5 m away) before the bleep on two consecutive occasions. No encouragement was provided by the researchers during this test. The ESWT was considered responsive to PR if the MCID fell between 170 and 209 seconds [12]. The same pre- and post-test measures were assessed as described above.

Sit-to-stand (STS) test

A modified Sit-To-Stand (STS) test (i.e., specific five-repetition variant [FTSTS]) was performed by participants, which assessed how quickly they could stand from sitting five times consecutively [14]. The MCID for this test following PR is an improvement of 1.7 seconds [15].

Dyspnoea

The Medical Research Council (MRC) dyspnoea scale was used to measure the level of breathlessness. It consists of grades ranging from 1 (none) to 5 (almost compete incapacity), with high grades indicating high perceived respiratory disability [16, 17]. The MRC dyspnoea scale is responsive to PR with estimated MCID of 1 point [18].

Psychological wellbeing

The Hospital Anxiety and Depression Scale (HADS) questionnaire is composed of 14 items with two 7-item subscales (HADS-A and HADS-D), both ranging from 0 to 21 [19]. Higher scores indicate more severe levels of psychological distress. The HADS is responsive to PR with estimated MCID of 2 points on each subscale [20].

Supplementary material G - Baseline characteristics

Characteristics	All participants (n=30)
**Duration of work over last 12 months, n (%)**	
Less than 6 months	1 (3.3)
6 months or more	8 (26.7)
**Current situation (if not employed), n (%)**	
Full-time homemaker or caregiver	13 (43.3)
Works in family business	0 (0.0)
Unemployed – able to work and seeking occupation	0 (0.0)
Unemployed – able to work and not seeking occupation	0 (0.0)
Unemployed – unable to work	0 (0.0)
Retired	8 (26.7)
**Lung health**	
Smoking status, n (%)	
Current	0 (0.0)
Former	6 (20.0)
Never	24 (80.0)
Biomass fuel exposure, n (%)	
Current	2 (6.7)
Former	0 (0.0)
Never	28 (93.3)
Pack years, mean (SD)	15.1 (12.4)
Secondary respiratory diagnosis, n (%)	
Asthma	1 (3.3)
Pneumonia	1 (3.3)
Post-tuberculosis	3 (10)
Pulmonary hypertension	4 (13.3)
Post-bronchodilator spirometry	
FEV_1 (L), mean (SD)	1.54 (0.55)
FVC (L), mean (SD)	1.88 (0.67)
FEV_1/FVC, mean (SD)	0.83 (0.08)
Hospitalisations in the last 12 months, n (%) with 1 or more	12 (40)
**Comorbidities, n (%)**	
Cardiac disease	6 (20.0)
Peripheral vascular disease	0 (0.0)
Hypertension	10 (33.3)
Diabetes	13 (43.3)
Kidney disease	3 (10.0)
Arthritis/MSK	3 (10.0)
Mental health disorder	0 (0.0)
Malignancy	0 (0.0)
Post-COVID	9 (30.0)
Hypothyroidism	2 (6.7)
Obstructive sleep apnoea hypopnoea syndrome	1 (3.3)
Osteoporosis/osteopenia	4 (13.3)
Gastroesophageal reflux disease	3 (10.0)
**Medication use, n (%)**	
ICS	0 (0.0)
LABA	3 (10.0)
LAMA	0 (0.0)
ICS/LABA	13 (43.3)
LABA/LAMA	0 (0.0)
SABA	4 (13.3)
SAMA	0 (0.0)

SABA/SAMA	0 (0.0)
LTRA	3 (10.0)
Triple therapy	1 (3.3)
Oxygen treatment, n (%)	
Long term oxygen therapy	4 (13.3)
ABOT	4 (13.3)
Short burst oxygen	15 (50.0)
Anti-histamines	14 (46.7)
Cough syrup	3 (10.0)
Mucolytics	16 (53.3)
Systemic steroids	20 (66.7)
Antibiotics	12 (40.0)
Antifibrotics	28 (93.3)
TB treatment	0 (0.0)

Supplementary material H - Secondary outcome results

Outcome measures	Pre-intervention (n=25)	Post-intervention (n=25)
MRC dyspnoea score, n (%)		
1	0 (0.0)	7 (28.0)
2	9 (36.0)	8 (32.0)
3	1 (4.0)	10 (40.0)
4	12 (48.0)	0 (0.0)
5	3 (12.0)	0 (0.0)
HADS Depression, n (%)		
Normal (0-7)	12 (48.0)	19 (76.0)
Mild (8-10)	7 (28.0)	5 (20.0)
Moderate (11-15)	6 (24.0)	1 (4.0)
Severe (16-21)	0 (0.0)	0 (0.0)
HADS Anxiety, n (%)		
Normal (0-7)	13 (52.0)	20 (80.0)
Mild (8-10)	5 (20.0)	3 (12.0)
Moderate (11-15)	5 (20.0)	2 (8.0)
Severe (16-21)	2 (8.0)	0 (0.0)
EQ-5D-5L mobility, n (%)		
No problems	2 (8.0)	11 (44.0)
Slight problems	8 (32.0)	7 (28.0)
Moderate problems	10 (40.0)	6 (24.0)
Severe problems	5 (20.0)	1 (4.0)
Unable to	0 (0.0)	0 (0.0)
EQ-5D-5L self-care, n (%)		
No problems	3 (12.0)	14 (56.0)
Slight problems	9 (36.0)	6 (24.0)
Moderate problems	11 (44.0)	4 (16.0)
Severe problems	2 (8.0)	1 (4.0)
Unable to	0 (0.0)	0 (0.0)
EQ-5D-5L usual activities, n (%)		
No problems	4 (16.0)	14 (56.0)
Slight problems	8 (32.0)	10 (40.0)
Moderate problems	7 (28.0)	0 (0.0)
Severe problems	6 (24.0)	1 (4.0)
Unable to	0 (0.0)	0 (0.0)
EQ-5D-5L pain/discomfort, n (%)		
No pain or discomfort	11 (44.0)	20 (80.0)

Slight pain or discomfort	9 (36.0)	5 (20.0)
Moderate pain or discomfort	5 (20.0)	0 (0.0)
Severe pain or discomfort	0 (0.0)	0 (0.0)
Extreme pain or discomfort	0 (0.0)	0 (0.0)
EQ-5D-5L anxiety/depression, n (%)		
Not anxious or depressed	3 (12.0)	12 (48.0)
Slightly anxious or depressed	8 (32.0)	9 (36.0)
Moderately anxious or depressed	12 (48.0)	4 (16.0)
Severely anxious or depressed	2 (8.0)	0 (0.0)
Extremely anxious or depressed	0 (0.0)	0 (0.0)

Note: *Data presented as mean (SD) unless otherwise stated. MRC, Medical Research Council; KBILD, King's Brief Interstitial Lung Disease; CAT, COPD Assessment Test; CCQ, Clinical COPD Questionnaire; HADS, Hospital Anxiety and Depression Scale; EQ-5D-5L, European Quality of Life 5-Dimensions*

Exercise capacity outcomes

Physical measures	Pre-intervention (n=20)	Post-intervention (n=20)
**Incremental shuttle walk test (ISWT)**		
Start SpO_2, %	96.8 (1.6)	97.2 (0.8)
End SpO_2, %	89.4 (6.4)	89.9 (5.5)
Start heart rate, beats/min	94 (15)	93 (14)
End heart rate, beats/min	120 (19)	126 (15)
Start Borg score	0.1 (0.2)	0.0 (0.1)
End Borg score	4.6 (2.3)	3.8 (1.5)
Start RPE score	6.0 (0.0)	6.0 (0.0)
End RPE score	12.7 (2.3)	11.7 (1.6)
Reason for termination, n (%)		
Shortness of breath	2 (10.0)	0 (0.0)
Leg fatigue	1 (5.0)	0 (0.0)
SpO_2	3 (15.0)	1 (5.0)
Timing	0 (0.0)	0 (0.0)
Unable to keep up with beeps	16 (80.0)	19 (95.0)
Other	0 (0.0)	0 (0.0)
**Endurance shuttle walk test (ESWT)**		
Speed, km/h	4.1 (0.9)	4.1 (0.9)
Level	8.9 (3.4)	8.9 (3.4)
End heart rate, beats/min	129 (18)	135 (22)
End SpO_2, %	87.8 (7.0)	88.5 (6.4)
End Borg score	5.5 (2.2)	4.8 (1.5)
End RPE score	13.3 (2.7)	12.4 (2.0)
Reason for termination, n (%)		
Shortness of breath	9 (45.0)	6 (30.0)
Leg fatigue	6 (30.0)	9 (45.0)
SpO_2	5 (25.0)	4 (20.0)
Timing	0 (0.0)	1 (5.0)
Other	0 (0.0)	0 (0.0)

Note: Data presented as mean (SD) unless otherwise stated.

References

1. Hanif H, Ahmed O, Manifield J, et al. Understanding the lived experience of idiopathic pulmonary fibrosis and how this shapes views on home-based pulmonary rehabilitation in Delhi, India. *Chronic Respiratory Disease* 2024; 21: 14799731241258216.
2. Sahasrabudhe SD, Orme MW, Jones AV, et al. Potential for integrating yoga within pulmonary rehabilitation and recommendations of reporting framework. *BMJ Open Respiratory Research* 2021; 8: e000966.
3. Group TE. EuroQol-a new facility for the measurement of health-related quality of life. *Health policy* 1990; 16: 199-208.
4. Nolan CM, Longworth L, Lord J, et al. The EQ-5D-5L health status questionnaire in COPD: validity, responsiveness and minimum important difference. *Thorax* 2016; 71: 493-500.
5. Patel AS, Siegert RJ, Brignall K, et al. The development and validation of the King's Brief Interstitial Lung Disease (K-BILD) health status questionnaire. *Thorax* 2012; 67: 804-810.
6. Nolan CM, Birring SS, Maddocks M, et al. King's Brief Interstitial Lung Disease questionnaire: responsiveness and minimum clinically important difference. *European Respiratory Journal* 2019; 54.
7. Van der Molen T, Willemse BW, Schokker S, et al. Development, validity and responsiveness of the Clinical COPD Questionnaire. *Health and quality of life outcomes* 2003; 1: 1-10.
8. Kon SS, Dilaver D, Mittal M, et al. The Clinical COPD Questionnaire: response to pulmonary rehabilitation and minimal clinically important difference. *Thorax* 2014; 69: 793-798.
9. Jones P, Harding G, Berry P, et al. Development and first validation of the COPD Assessment Test. *European Respiratory Journal* 2009; 34: 648-654.
10. Kon SS, Canavan JL, Jones SE, et al. Minimum clinically important difference for the COPD Assessment Test: a prospective analysis. *The lancet Respiratory medicine* 2014; 2: 195-203.
11. Singh SJ, Morgan M, Scott S, et al. Development of a shuttle walking test of disability in patients with chronic airways obstruction. *Thorax* 1992; 47: 1019-1024.
12. Zatloukal J, Houchen-Wolloff L, Ward S, et al. MID of shuttle walk tests in patients with ILD following pulmonary rehabilitation. *ERJ Open Research* 2025.
13. Revill S, Morgan M, Singh S, et al. The endurance shuttle walk: a new field test for the assessment of endurance capacity in chronic obstructive pulmonary disease. *Thorax* 1999; 54: 213-222.
14. Bohannon RW. Sit-to-stand test for measuring performance of lower extremity muscles. *Perceptual and motor skills* 1995; 80: 163-166.
15. Jones SE, Kon SS, Canavan JL, et al. The five-repetition sit-to-stand test as a functional outcome measure in COPD. *Thorax* 2013; 68: 1015-1020.
16. Bestall J, Paul E, Garrod R, et al. Usefulness of the Medical Research Council (MRC) dyspnoea scale as a measure of disability in patients with chronic obstructive pulmonary disease. *Thorax* 1999; 54: 581-586.
17. Stenton C. The MRC breathlessness scale. *Occupational Medicine* 2008; 58: 226-227.
18. Crisafulli E and Clini EM. Measures of dyspnea in pulmonary rehabilitation. *Multidisciplinary respiratory medicine* 2010; 5: 1-9.
19. Zigmond AS and Snaith RP. The hospital anxiety and depression scale. *Acta psychiatrica scandinavica* 1983; 67: 361-370.
20. Smid DE, Franssen FM, Houben-Wilke S, et al. Responsiveness and MCID estimates for CAT, CCQ, and HADS in patients with COPD undergoing pulmonary rehabilitation: a prospective analysis. *Journal of the American Medical Directors Association* 2017; 18: 53-58.
